# Making Air Pollution Visible: A Tool for Promoting Environmental Health Literacy

**DOI:** 10.2196/publichealth.7492

**Published:** 2017-04-12

**Authors:** Ekaterina Galkina Cleary, Allison P Patton, Hsin-Ching Wu, Alan Xie, Joseph Stubblefield, William Mass, Georges Grinstein, Susan Koch-Weser, Doug Brugge, Carolyn Wong

**Affiliations:** ^1^ Center for Integration of Science and Industry Bentley University Waltham, MA United States; ^2^ Environmental and Occupational Health Sciences Institute Rutgers University Piscataway, NJ United States; ^3^ Department of Public Policy and Public Affairs University of Massachusetts Boston Boston, MA United States; ^4^ Institute for Asian American Studies University of Massachusetts Boston Boston, MA United States; ^5^ Computer Science Department University of Massachusetts Lowell Lowell, MA United States; ^6^ Center for Data Science University of Massachusetts Amherst Amherst, MA United States; ^7^ Department of Public Health and Community Medicine Tufts University School of Medicine Boston, MA United States; ^8^ Department of Civil and Environmental Engineering Tufts University Medford, MA United States; ^9^ Tisch College of Civic Life Tufts University Medford, MA United States

**Keywords:** computer visualization, digital cartography, environmental health literacy, health communication, environmental health, computer-based education, air pollution, ultrafine particles, immigrant education

## Abstract

**Background:**

Digital maps are instrumental in conveying information about environmental hazards geographically. For laypersons, computer-based maps can serve as tools to promote environmental health literacy about invisible traffic-related air pollution and ultrafine particles. Concentrations of these pollutants are higher near major roadways and increasingly linked to adverse health effects. Interactive computer maps provide visualizations that can allow users to build mental models of the spatial distribution of ultrafine particles in a community and learn about the risk of exposure in a geographic context.

**Objective:**

The objective of this work was to develop a new software tool appropriate for educating members of the Boston Chinatown community (Boston, MA, USA) about the nature and potential health risks of traffic-related air pollution. The tool, the Interactive Map of Chinatown Traffic Pollution (“Air Pollution Map” hereafter), is a prototype that can be adapted for the purpose of educating community members across a range of socioeconomic contexts.

**Methods:**

We built the educational visualization tool on the open source Weave software platform. We designed the tool as the centerpiece of a multimodal and intergenerational educational intervention about the health risk of traffic-related air pollution. We used a previously published fine resolution (20 m) hourly land-use regression model of ultrafine particles as the algorithm for predicting pollution levels and applied it to one neighborhood, Boston Chinatown. In designing the map, we consulted community experts to help customize the user interface to communication styles prevalent in the target community.

**Results:**

The product is a map that displays ultrafine particulate concentrations averaged across census blocks using a color gradation from white to dark red. The interactive features allow users to explore and learn how changing meteorological conditions and traffic volume influence ultrafine particle concentrations. Users can also select from multiple map layers, such as a street map or satellite view. The map legends and labels are available in both Chinese and English, and are thus accessible to immigrants and residents with proficiency in either language. The map can be either Web or desktop based.

**Conclusions:**

The Air Pollution Map incorporates relevant language and landmarks to make complex scientific information about ultrafine particles accessible to members of the Boston Chinatown community. In future work, we will test the map in an educational intervention that features intergenerational colearning and the use of supplementary multimedia presentations.

## Introduction

There is a pressing need for new tools to promote environmental health literacy [1]. As research reveals new knowledge about the public health effects of environmental pollutants, it remains challenging to communicate complex scientific information to laypersons. Environmental health literacy will be significantly improved when people not only learn factual knowledge but also understand what tactics can reduce their exposure to risk [1]. Recent advances in the technology of computer mapping hold the potential to increase environmental health literacy by helping users visualize information about environmental hazards in spatial terms and within a geographic area. By forming mental representations of this information, users may be able to make more informed decisions and have more well-defined discussions when considering health-promoting measures [2].

This work aimed to develop a computer visualization tool to promote environmental health literacy about traffic-related pollution in Boston Chinatown, Massachusetts, USA. One component of traffic pollution consists of particulate matter. Particulate matter in ambient air is a leading cause of mortality and morbidity worldwide. It is estimated that ambient particulate matter, which consists of particles 2.5 μm or less in diameter (PM2.5), causes 100,000 to 200,000 deaths in the United States and 3.2 million deaths globally each year [3-5]. Ultrafine particles, a subfraction of PM2.5 that are less than 0.1 μm in diameter, tend to be higher locally near heavy traffic. Thus, a community’s proximity to highways and major roadways presents a health risk to its inhabitants [6,7]. In Chinatown, where poverty levels are higher than those in most other parts of the city, many residents have low levels of educational attainment and limited English proficiency [8]. For this reason, it is important to improve residents’ access to health information.

People vary in their capacity to access, learn, and use information [9]. Efforts to disseminate health information often result in differential learning, with the poor and socially disadvantaged benefiting the least [10,11]. Messages about environmental health need to be tailored to audiences with varying socioeconomic and ethnic backgrounds [1]. Extensive research points to the importance of making computer tools accessible to individuals with low levels of English proficiency and limited computer skills [12]. Development of accessible tools for improving environmental health literacy would help educators cross these linguistic and technological barriers.

Pictures can aid the accessibility of health communication messages [13]. Studies have shown that visual information is better retained than verbal information by users in the cognitive process of building mental models [14-17]. Because of this, visualization technology is particularly well-suited to education about ultrafine particles, as the small size of these particles makes them invisible to the naked eye, and people are often unaware of their presence. Consequently, visualization of such ultrafine particle concentrations on a computer screen provides a means of increasing community awareness.

Visualization tools can also aid community health education. For example, computer maps can offer multiple views of a geographic area and have the potential to improve cognitive performance. The main bottleneck in cognitive processing and visual thinking is often the limited capacity of working memory, which can retain only about 3 to 5 objects [18]. Having access to more than one visualization can allow users to store information in working memory while thinking about, analyzing, and processing that information, and can improve learning and decision making [19]. In a map of pollution, for example, one map layer might show selected features of the built environment, such as roadways and high-rise buildings. Another layer can depict the location and extent of open green space or vegetation. In addition, interactivity of digital maps can further facilitate learning [20]. Users can manipulate the display through visual animations that convey different types of information. This would allow them to explore the effect of temperature and wind severity, and thereby increase the potential for interest and engagement.

Visualization additionally helps overcome the limitations of health literacy promotion reliant on textual representation of information, which often simplifies concepts for readability [21]. Simplification can erase nuances important to comprehension of complex scientific information in general and, in our case, about ultrafine particles. A map showing the spatial distribution of the particles in a community can improve awareness of many factors in the physical environment that affect community residents’ daily lives, such as roadways, green space, and residential buildings. As environmental researchers discover how changes in concentrations of ultrafine particles in the air are affected by factors such as temperature and wind, it becomes possible to depict these changing concentrations by means of animated visualizations.

The objective of this work was to develop a visualization tool that would educate members of the Boston Chinatown community about one form of traffic-related pollution, ultrafine particles. This paper focuses on development of the Interactive Map of Chinatown Traffic Pollution (or Air Pollution Map). Its use in educational interventions will be discussed in a future publication.

## Methods

We designed the Air Pollution Map as the first step of a community educational intervention to meet the challenges of disseminating complex scientific knowledge about environmental hazards to laypersons. Users can explore spatial patterns of ultrafine particle concentrations, measured in particle number concentration (PNC) units, and see how concentrations vary with changing conditions, including temperature, wind severity, wind direction, and traffic volume. The map we created provides 4 visual controls as sliders, which allow a user to modify conditions of temperature, wind direction, wind speed, and traffic volume (model explained below). We created bilingual English-Chinese Web-based and desktop-based versions of the visualization.

We created the Air Pollution Map using Weave v1.9.38, a Web-based analysis and visualization environment [22]. Weave was developed at the University of Massachusetts Lowell, Lowell, MA, USA and is now supported as an open source tool. Weave provides the ability to integrate, analyze, and visualize data using a suite of over 20 different interactive tools. Completed visualizations can be disseminated via a website.

Weave [23] is freely available to the public and is simple to install on personal computers. Its accessibility facilitates its use by educators in community settings such as schools, community-based organizations, libraries, and homes. The interactivity of the tool that we developed in Weave is designed to be used as a launching point for group-based conversation about the problems posed by air pollution and tactics that communities can pursue to reduce exposure.

The data used to construct the Air Pollution Map were collected and analyzed by an investigatory team of the Community Assessment of Freeway Exposure and Health Study (CAFEH). CAFEH measured ultrafine particle concentrations under a variety of weather and traffic conditions in Boston Chinatown [24] and built a statistical model that describes how the ambient conditions were related to the ultrafine particle concentrations [25]. We applied this model as an algorithm and incorporated it into the interactive map in ways that we hoped would allow users to interactively explore hypothetical scenarios that affect ultrafine particle concentrations across the community.

**Figure 1:**
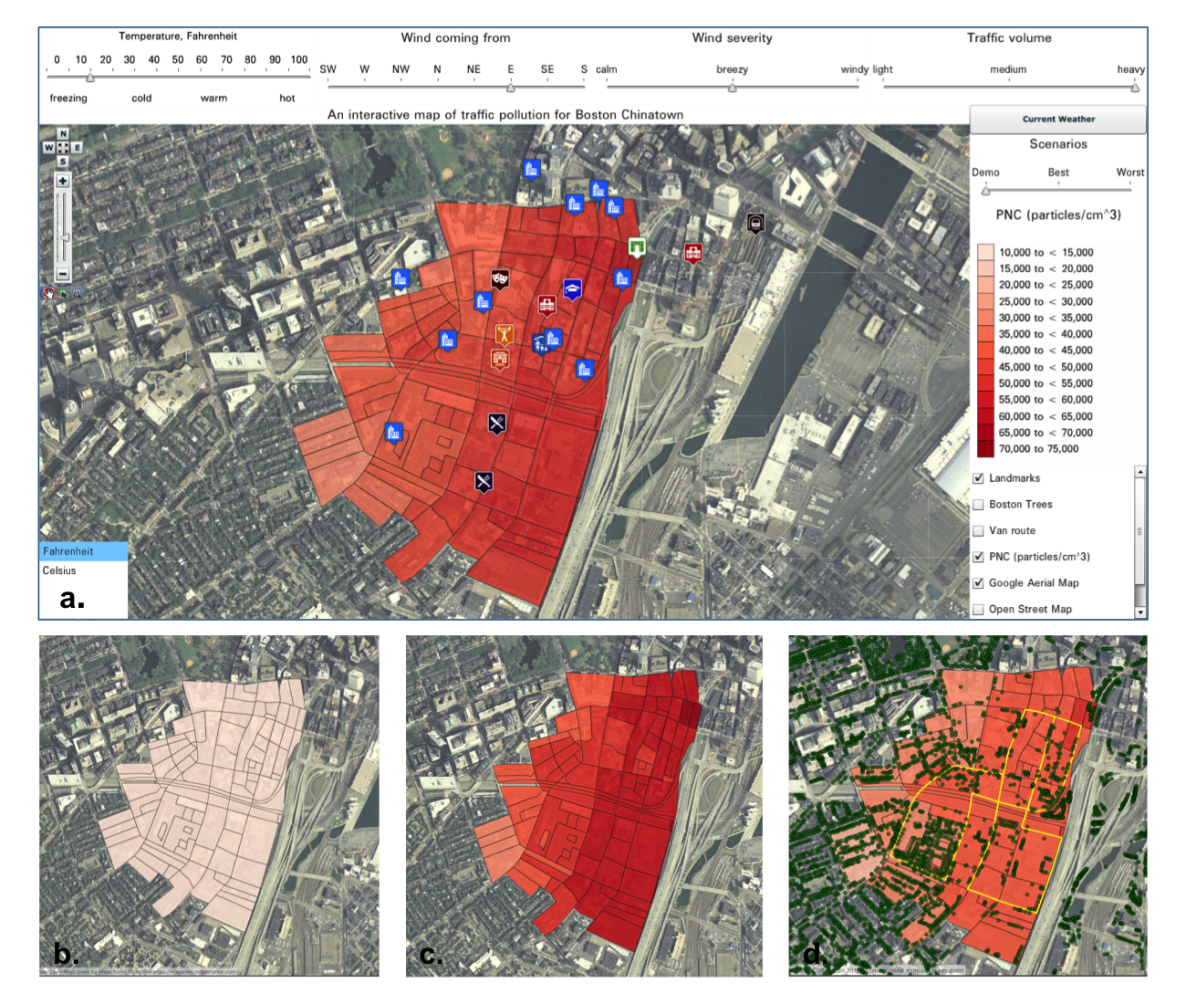
Several views of the Interactive Map of Chinatown Traffic Pollution: (a) Full aerial Google maps view showing sliders, map layers, scenario slider, and color legend; (b) scenario of the lowest ultrafine particle concentrations; (c) scenario of the highest ultrafine particle concentrations; (d) Community Assessment of Freeway Exposure and Health Study mobile laboratory route (solid line) taken to measure ultrafine particles and locations of surrounding trees (dots). Visualizations created using Weave software v1.9.38. PNC: particle number concentration.

The variables included in our algorithm were wind speed, temperature, traffic volume, and wind direction. These were the primary predictive variables in the ultrafine particles model (Table 1). The model predicts lower ultrafine particle concentrations when winds are stronger, which blow the particles away more effectively. Higher concentrations are expected when there are more cars traveling on Interstate 93, a major north-south highway near Boston Chinatown, resulting in increased emissions. Concentrations are also higher with lower temperatures due to (1) more formation of particles from less-efficient engines, (2) less dispersion of particles in the air, and (3) lower particle removal due to slower evaporation and particles sticking together. The model predicts that when the wind comes from the east, concentrations are highest near Interstate 93, the major highway, and a major train station on the far side of the highway (see Figure 1). Under these conditions, concentrations decrease toward the west side of Chinatown. We developed and ran the model in the R-Project v3.1.1 programming language (R Core Team, R Foundation for Statistical Computing), and we averaged PNC to census blocks for use in our map.

We consulted community experts to assist in developing the user interface for the map. Two community health promoters who had prior experience in educational projects concerning traffic pollution in Chinatown recommended Chinese wording for menu labels and selected community landmarks recognizable to residents.

The University of Massachusetts Institutional Review Board approved this study (#2014161).

**Table 1:** Variables of interest and their effects on particle number concentration (PNC).

Variable	Effect of increase in variable	Selectable values in Weave
Wind speed	Percentage decrease in PNC	Calm, breezy, windy
Temperature	Percentage decrease in PNC	0 to 100°F or –20 to 40°C
Traffic volume	Percentage increase in PNC	Light, medium, heavy
Wind direction	More complicated: highest for east winds, and with larger effect near Interstate 93	N, NE, E, SE, S, SW, W, NW^a^

^a^Wind direction variables: north (N), northeast (NE), east (E), southeast (SE), south (S), southwest (SW), west (W), northwest (NW).

## Results

The Air Pollution Map (Figure 1) allows users to see how meteorological and traffic conditions can affect changes in ultrafine particle concentration by moving the 4 sliders for temperature, wind direction, wind severity, and traffic volume (Figure 1 a). PNC at each census block is shown through choropleth mapping, in which brighter color intensity indicates higher pollution levels (key in Figure 1 a). We chose the wording used for the labels on the sliders to conform to popular language conventions among members of the Chinatown community. For example, “calm,” “breezy,” and “windy” are commonly understood terms describing wind severity. Icons for commonly recognized landmarks are used to orient the user within the neighborhood.

There are several layers to the map that can be made visible or hidden. These include a base map showing the street level or a satellite view of the study area with prominent landmarks (Figure 1 a), the route taken by the CAFEH mobile laboratory when measuring ultrafine particles (Figure 1 d), the location of trees (Figure 1 d), and the ultrafine particle concentration in particles/cm^3^ (Figure 1 b and 1c). These features allow users to learn experientially by changing values for the variables, such as setting the weather conditions to current values. In doing so, they can discover which combination of conditions leads to the highest (Figure 1 c) and lowest (Figure 1 b) concentrations of ultrafine particles. They can also explore effects of each of the input parameters while holding others constant or in interaction with one another. We included a separate “scenario” slider to quickly navigate to the conditions producing the best and worst levels of pollution (Figure 1 a). Additional exploratory map features include the ability to drill down to Chinatown street levels or zoom out to see adjacent neighborhoods.

Advanced toolbar features of Weave are purposefully hidden, so that buttons not vital to the educational process are not accessible to the novice user. The educational version of the map also hides the scenario slider, so that students may independently arrive at the contributors to the best and worst pollution levels by exploration. Weave’s session state-based architecture allows all significant interactions made with the system to be recorded (eg, selecting, subsetting, and toggling). By analyzing or even simply replaying the actions each student took while exploring the map, educators may find patterns that are informative about the learning process. For example, teachers can analyze steps students take to identify what combinations of temperature and wind conditions are associated with high levels of pollution.

The bilingual feature and the dual temperature scale (Celsius/Fahrenheit) were necessary to accommodate the needs of the different segments of the community, including longtime residents and newly arrived immigrants. The visualization tool was used in educational workshops targeted to high school youth, English-language adult learners, and seniors in Boston Chinatown. We developed a plan for evaluation of the educational intervention. In future work, we will analyze data collected on the effectiveness of the intervention and produce a report of findings.

A stable version of the Interactive Map of Chinatown Traffic Pollution is available [26]. The bilingual desktop version is available free of charge from the first or last author upon request.

## Discussion

Our Air Pollution Map aimed to make complex information about the spatial distribution of ultrafine particulate pollution in a community accessible and usable for residents of Boston Chinatown and to aid them in engaging in health-promoting behaviors. Health educators could, for example, use the visualizations to explain why altering certain choices, such as time and place of outdoor exercise, can reduce exposure [20]. Interactive maps are commonly used by professionals for environmental analysis and planning. However, user interfaces are rarely linguistically tailored to persons with limited English proficiency. One study has shown that limited English proficiency is associated with low health literacy across diverse racial groups. Among a population-based sample of limited English-proficiency adults, a higher percentage of Chinese respondents self-reported lower health literacy (68.3%) compared with their white counterparts (18.8%) [27]. Increasing accessibility of information is an important part of reducing the digital divide in health literacy [28].

We sought to develop the Air Pollution Map with several features not offered in existing mapping tools. Unlike other air pollution maps, which focus on other pollutants, our tool makes ultrafine particles visible to the user. Moreover, concentrations of ultrafine particles are displayed at the census block level, an appropriate granularity of geographic unit for education about how concentrations of ultrafine particles vary across a neighborhood. We created the user interface for ease of use by individuals with limited English and technical skills. Specifically, the design approach emphasized accessibility by members of the Chinatown community, most of whom are immigrants. Map labels enable users of the tool who live and work in this neighborhood to recognize familiar streets or areas, including highways and local landmarks.

These features of the Air Pollution Map add new capabilities to an evolving technology of mapping air pollutants. Two other tools, Plume Air Report [29] and AirNow [30], display real-time information on pollution and provide recommendations for outdoor activities. The Community LINE Source Model (C-LINE) maps contributions of mobile sources to local air pollution under user-defined traffic emissions and meteorology conditions [31]. Each of these existing tools gives users access to important information about types of air pollution in their community, but not exposure to ultrafine particulate pollution.

In designing an educational intervention using the Air Pollution Map, it is important to consider not only the educational potential of the map but also its limitations. The present version requires users to enter values for temperature, wind severity and direction, and traffic volume by manipulating sliders. In future work, we will add a new capability for the user to automatically import the actual wind and temperature pattern for the current day and time. In addition, the user interface currently uses only English and Chinese. Translations to other languages such as Spanish would be helpful in order to widen accessibility to residents of other neighborhoods. Alterations of the user interface have not yet been implemented for persons who are visually impaired.

The next phase of work entails development and evaluation of an educational intervention using the Air Pollution Map. We have developed multimedia educational presentations, which have been shown to complement visual learning [32,33]. We also devised small-group learning activities and walking tours in the community to aid learning about sources of traffic-related pollution and features of the built environment. These multimodal learning activities are intended to take place in a supportive social environment, which helps motivate learning [34]. In particular, the educational approach emphasizes intergenerational colearning; specifically, teenagers who are familiar with computer technology are first trained to use the interactive map; in turn, the youth teach those who are less familiar with computer technology, such as elders.

## Abbreviations

CAFEH: Community Assessment of Freeway Exposure and Health Study
PM2.5: particulate matter ≤2.5 μm in diameter
PNC: particle number concentration
