# Reference Correction to: Making Air Pollution Visible: A Tool for Promoting Environmental Health Literacy

**DOI:** 10.2196/publichealth.8750

**Published:** 2017-12-20

**Authors:** Ekaterina Galkina Cleary, Allison P Patton, Hsin-Ching Wu, Alan Xie, Joseph Stubblefield, William Mass, Georges Grinstein, Susan Koch-Weser, Doug Brugge, Carolyn Wong

**Affiliations:** ^1^ Center for Integration of Science and Industry Bentley University Waltham, MA United States; ^2^ Environmental and Occupational Health Sciences Institute Rutgers University Piscataway, NJ United States; ^3^ Department of Public Policy and Public Affairs University of Massachusetts Boston Boston, MA United States; ^4^ Institute for Asian American Studies University of Massachusetts Boston Boston, MA United States; ^5^ Computer Science Department University of Massachusetts Lowell Lowell, MA United States; ^6^ Center for Data Science University of Massachusetts Amherst Amherst, MA United States; ^7^ Department of Public Health and Community Medicine Tufts University School of Medicine Boston, MA United States; ^8^ Department of Civil and Environmental Engineering Tufts University Medford, MA United States; ^9^ Tisch College of Civic Life Tufts University Medford, MA United States

The authors of “Making Air Pollution Visible: A Tool for Promoting Environmental Health Literacy” (JMIR Public Health Surveill 2017;3(2):e16) would like to make a correction to the text in Reference 26. It should read:

26. An interactive map of traffic pollution for Boston Chinatown. URL: http://chinatown.oicweave.com [accessed 2017-08-14] [WebCite Cache ID 6siTaGLNk]

The corrected article will appear in the online version of the paper on the JMIR website on December 20, 2017, together with the publication of this correction notice. Because this was made after submission to PubMed Central, the corrected article also has been re-submitted to PubMed Central.

